# Age and gender differences in the association between social participation and instrumental activities of daily living among community-dwelling elderly

**DOI:** 10.1186/s12877-017-0491-7

**Published:** 2017-04-28

**Authors:** Kimiko Tomioka, Norio Kurumatani, Hiroshi Hosoi

**Affiliations:** Nara Prefectural Health Research Center, Nara Medical University, Shijo-cho 840, Kashihara city, Nara 634-8521 Japan

**Keywords:** Social participation, Instrumental activities of daily living, Paid work, Elderly, Age differences, Gender differences

## Abstract

**Background:**

Although many studies have suggested social participation (SP) has beneficial effects on elderly people’s health, most of them failed to deal with paid work. Additionally, few studies have focused on the age effect between SP and older people’s health. To investigate whether the association between SP, including paid work, and instrumental activities of daily living (IADL), exhibits not only in gender, but also in age among community-dwelling older adults.

**Methods:**

In 2014, we distributed self-administered questionnaires to all community-dwelling elderly aged ≥65 in two medium-sized cities in Nara Prefecture, Japan (*n* = 32,825). 22,845 residents submitted the questionnaire (response rate, 69.6%). Analyzed subjects were limited to 17,680 persons who had neither dependency in basic ADL nor missing data for required items. SP was assessed based on participation frequency in seven types of social activities: volunteer groups, sports groups, hobby groups, cultural groups, senior citizens’ clubs, neighborhood community associations, and paid work. Using Poisson regression models, prevalence ratio for poor IADL was calculated. To examine age and gender differences in the association between SP and IADL, we performed stratified analyses by age and gender group; male young-old (aged 65–74), male old-old (aged ≥75), female young-old, and female old-old.

**Results:**

Prevalence of those with poor IADL was 17.1% in males and 4.5% in females, showing a significant gender difference. After adjustment for relevant covariates, volunteer groups were inversely associated with poor IADL only in males and the relationship was stronger in the old-old group than in the young-old group. Conversely, only females had a significant inverse association between paid work and poor IADL, and the association was not reliant on their ages but only those who participated infrequently had a favorable effect. Influence of age in the beneficial association between SP and IADL was generally larger in the old-old group than in the young-old group, but hobby groups were inversely associated with poor IADL, regardless of age, gender, and frequency.

**Conclusions:**

Our results suggest that SP in older age is positively associated with IADL, however, the association seems to differ depending on the type of activities participated in, the participants’ gender, and their age.

**Electronic supplementary material:**

The online version of this article (doi:10.1186/s12877-017-0491-7) contains supplementary material, which is available to authorized users.

## Background

Elderly people’s social participation (SP) is believed to be a vital key in their active aging [1]. Many previous studies have reported that elderly people’s SP has a positive impact on their physical and mental health including longevity [2, 3], sustention of ADL [2, 4] and cognitive function [5, 6], higher level of health-related quality of life [7], and prevention of depression [8]. On the other hand, it has been pointed out that SP’s impact on elderly people’s health may change depending on its content [2, 3, 9] and the type of activities participated in [4–6]. Frequent SP can make the elderly feel obligated and may have a negative impact on their health [10, 11]. These issues suggest that rather than trying to grasp the health impact of all SP activities on elderly people’s health in a comprehensive manner, it is necessary to pay attention to the type of SP and the frequency of participation.

To spend everyday community life in an autonomous and active manner, elderly people require a higher level of living function than they need to conduct basic activities of daily living (BADL), the ability required to live autonomously while taking care of one’s self [12]. One of the indexes of higher living function is instrumental activities of daily living (IADL), which is having the ability to use public transportation, prepare meals, shop, and manage their own finances [12]. Because it’s noted that there are gender differences in IADL difficulties among older adults [13], it is necessary to examine male and female IADL separately when viewing it as a health outcome. Since there are limited numbers of studies regarding the association between SP and IADL, we examined the types of social activities which community dwelling elderly people participate in as well as the impact of SP on IADL with specific attention given to gender difference [14, 15]. Their results were as follows; a prospective cohort study [14] revealed that among females, participation in volunteer groups, hobby groups, senior citizen clubs, and local events was associated with decreased risk of IADL decline, whereas among males, only participation in hobby groups was protective for IADL decline. A cross-sectional study [15] revealed that among females, a significant inverse association between frequent participation and poor IADL was observed for all types of social groups, whereas among males, the association was limited to sports groups and senior citizens’ clubs. These observed results revealed that females received a stronger impact from SP than males as well as social activities showing that significant relationships with IADL are different on each gender.

Prior studies have pointed out that the elderly receive a greater impact from SP on health than younger individuals, and that risk and protective factors for functional decline differ between the younger-old and the older-old [16]. Although there are several studies regarding SP of community dwelling elderly people with focus given to gender differences [2, 6, 8, 9], there are very few studies giving attention to the impact of age at the time of SP [5]. In our previous studies, too, age was only used as a covariate [14, 15]. As the rate of those who participate in social activities and those who maintain IADL decrease along with aging [17, 18], we consider the examination of age difference in association between SP and IADL to be essential. Furthermore, our previous studies [14, 15] didn’t consider “paid work” as one of the most important SPs for elderly people [1, 19]. In countries like Japan, where an aging population combined with a diminishing number of children is progressing, employment of the elderly is an important issue in securing manpower [20]. Therefore, it is necessary to examine the relationship between IADL, which is one of the indexes of healthy longevity, and the elderly population’s paid work on each gender and age group.

In this study, based on the questionnaire data collected from all community dwelling elderly people, the researchers try to reveal how types and frequencies of SP, including paid work, impact the IADL of different genders and age groups.

## Methods

### Study population

The field of this survey’s participants is two medium-sized cities in Nara Prefecture, Japan. Both cities are located in the mid-west part of Nara Prefecture and have developed as residential areas as they share a border with a major urban area, Osaka Prefecture. Table 1 shows the basic information of both cities. Compared to the population aging rate in Japan, 26.0% as of October 2014, one can say A City’s population aging rate is similar, but that B City’s is lower. In March 2014, questionnaires were sent to all community-dwelling elderly aged 65 and over (*n* = 32,825, except those living in nursing homes) and collected by regular mail.

**Table 1:** Basic information on the surveyed municipalities

	A City	B City
Total population as of October 2014	68,168	78,273
Population density as of October 2014 (person/km^2^)	4136.4	3226.4
Population aging rate as of October 2014	26.7%	20.6%
No. of distributed questionnaires	17,615	15,210
No. of submitted questionnaires	11,870	10,975
Response rate	67.4%	72.2%
No. of subjects with dependent BADL	1949	1565
No. of subjects with missing data on BADL, IADL, and/or SP	984	667
No. of analytical subjects	8937	8743
Age of analytical subjects (mean, median, range)	72.9, 72.0, 65–98	72.9, 72.0, 65–100
Male prevalence of analytical subjects	45.7%	47.6%

*BADL* basic activities of daily living, *IADL* instrumental activities of daily living, *SP* social participation

All study participants provided signed informed consent. This study protocol was approved by the Nara Medical University Ethics Committee (approval number 990).

### Assessment of instrumental activities of daily living (IADL)

The Tokyo Metropolitan Institute of Gerontology Index of Competence (TMIG-IC) [21] was used to evaluate IADL. TMIG-IC (see Additional file 1) is a self-administered questionnaire to evaluate aged people’s higher-level functional capacity and is comprised of 3 subscales. One of the subscales, IADL, is comprised of 5 items. When a respondent replies that they can independently conduct all 5 items, we determined the person to be IADL self-dependent. Those ones who claimed they couldn’t conduct any of the items were determined to have poor IADL [22].

### Assessment of social participation (SP)

Besides the six types used in a previous study [15] (i.e., volunteer groups, sports groups, hobby groups, cultural groups, senior citizens’ clubs, and neighborhood community associations), paid work was adopted. We requested subjects to select a degree of participation frequency on each of seven types of SP from “4 times or more per week,” “2 to 3 times per week,” “once a week (i.e., weekly),” “several times per month (i.e., monthly),” “several times per year (i.e., yearly),” and “not participating”. See Additional file 2 for the analysis of responses regarding the frequency of participation in each SP. We set the participation frequency of those who belong to social groups to be “frequent participants” if they participated weekly or more and “infrequent participants” if they participated monthly or less, which divided the participants roughly in half. However, there were very few “weekly or more” responses for senior citizens’ clubs, a majority of participants in neighborhood community associations attended the activities yearly, and the majority of those who attended paid work participated 4 times or more per week. Additionally, there were no participants with poor IADL who participated in senior citizens’ clubs weekly or more among females aged 65–74, in neighborhood community associations weekly or more among both females aged 65–74 and females aged 75 over, and in paid work monthly or less among females aged 65–74 (see Additional file 3). Therefore, we adjusted the participation frequencies definitions for those 3 activities. Regarding senior citizens’ clubs and neighborhood community associations, participating monthly or more was defined as frequent participation and yearly was defined as infrequent participation. Regarding paid work, participating 4 times or more per week was defined as frequent participation, while 3 times or less per week was defined as infrequent participation.

### Covariates

Based on prior studies [4–9, 14, 15, 23–25], the following variables were used as covariates that may mediate the association between SP and IADL: age, residential area, marital status, subjective economic situations, pensions, chronic diseases, body mass index (BMI), alcohol, smoking, dietary habit, depression, cognitive function, self-rated health, and having a purpose in life. Data on age and residential areas were provided by city governments, and data for other covariates were procured from the questionnaire.

Regarding chronic diseases, the authors requested respondents to reveal the diseases they were under treatment for. Diseases disclosed include hypertension, diabetes mellitus, heart disease, cerebrovascular disease, cancer, chronic respiratory disease, digestive system disease, urogenital disease, musculoskeletal disorders, otological disease, and ophthalmologic disease. The prevalence of each disease is shown in Additional file 4. The top 3 diseases in prevalence (i.e., hypertension, ophthalmologic disease, and musculoskeletal disorders) were identified as prevalent comorbidities in community-dwelling elderly, and used as indicators of chronic diseases. Depression was assessed using the 5-item short form of the Geriatric Depression Scale [26] (score range 0–5), and a score of ≥2 was judged as having depression. Cognitive function was assessed using the Cognitive Performance Scale [27] (score range 0–6), and a score of ≥1 was classified as poor cognitive function. Self-rated health and having a purpose in life were evaluated to assess quality of life. Self-rated health was assessed by asking a single question “How is your health in general? Is it very good, good, poor, or very poor?” Having a purpose in life was also assessed by asking a single question “Do you have a sense of purpose in life? Yes or no?” Prior studies have used self-rated health and a sense of purpose in life as assessments of quality of life of the elderly, and reported that both are associated with a decline in IADL among community-dwelling elderly [24, 25].

We set all covariates except age as dummy variables. According to the statistical recommendation for handling missing covariates [28], those with missing values were set as a missing category in each covariate. See Additional file 4 for the categories of each item and distribution of answers.

### Statistical analyses

We conducted the sample size approximation using Epi Info version 7.2, and confirmed that our study population had an adequate design of needed sample size. The details of sample size approximation are provided in Additional file 5.

To investigate the association between SP and IADL, we used Poisson regression analysis with robust variance estimator. Prevalence ratio (PR) of SP for poor IADL was calculated with reference to those with non-participation in each activity, and with adjustment for all covariates. To examine age and gender differences in the association between SP and IADL, we performed stratified analyses by age and gender group; male young-old (aged 65–74), male old-old (aged ≥75), female young-old, and female old-old. The level of significance was 0.05 (two-tailed). We conducted statistical analyses using IBM SPSS Statistics version 24.0.

## Results

**Fig. 1:**
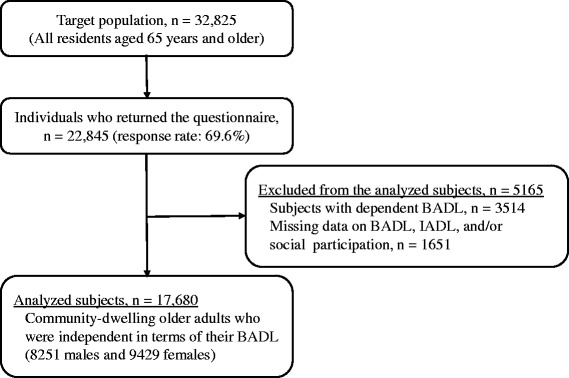
Flow chart of study participants. *BADL* basic activities of daily living, * IADL* instrumental activities of daily living

Figure 1 displays a flow chart of study participants. Among the target population (*n* = 32,825), 22,845 people returned the questionnaire, which makes the response rate 69.6%. See Additional file 6 for the basic attributes of those who submitted questionnaires. Responses showed a tendency that the higher the age, the lower the submission rate. Also, those who required higher nursing care needs were less likely to submit the questionnaire. There was no difference in the response rate between males and females. Disability in BADL does not only restrict SP but relates to limitations in IADL [12, 23], therefore, we eliminated 3514 respondents whom we determined were not self-reliant in BADL. We defined elderly people without BADL self-reliance as those who require nursing care under the public long-term care insurance system and/or those who answered that they needed assistance for at least one of the 5 activities listed on the questionnaire (eating, going to the toilet, bathing, dressing, and walking indoors). Additionally, we eliminated 1651 respondents who did not answer one or more questions regarding BADL, IADL and SP as being not analyzable. After eliminating 5165 respondents, the number of subjects included in the final analyses (hereinafter referred to as analyzed subjects) was 17,680 (8251 males and 9429 females). There were no age differences among analyzed subjects between the 2 cities (Mann-Whitney test, *p* = 0.897), but B City’s rate of males was higher than that of A City (Fisher’s exact test, *p* = 0.011) (shown in the bottom of Table 1). Regarding the prevalence of poor IADL, it was 17.1% in males and 4.5% in females, showing that males had a significantly poorer IADL than females (Fisher’s exact test, *p* < 0.001).

Table 2 shows the age-stratified characteristics of study participants. A prevalence of those who were not married, had more chronic diseases, were underweight, suffered from depression, had poor cognitive function, poor self-rated health, no sense of purpose in life, and poor IADL increased as they became older (Cochran-Armitage test, *p* < 0.001). On the other hand, the prevalence of those who had poor subjective economic situations, were daily drinker, and were current smokers decreased as they became older (Cochran-Armitage test, *p* < 0.001). Regarding SP, a prevalence of participants in sports groups, neighborhood community associations, and paid work decreased as they became older, while more people attended senior citizens’ clubs as they aged (Cochran-Armitage test, *p* < 0.001). Participation in volunteer groups and hobby groups was not affected by participants’ ages.

**Table 2:** Characteristics by age category among the study participants (*n* = 17,680)

	65–68 years	69–71 years	72–76 years	77–100 years	*P* for trend^a^
	(*n* = 4811)	(*n* = 3424)	(*n* = 4811)	(*n* = 4634)	
Residential area: A City	50.1%	50.4%	50.7%	50.9%	0.409
Gender: males	45.7%	46.7%	47.9%	46.3%	0.413
Marital status: not married	19.3%	20.8%	24.8%	40.5%	<0.001
Subjective economic situations: poor	18.0%	17.9%	18.9%	14.2%	<0.001
Pensions: National pension	34.5%	36.4%	37.8%	37.6%	0.001
Presence of hypertension	34.8%	38.2%	40.8%	43.6%	<0.001
Presence of ophthalmologic disease	10.5%	14.5%	17.1%	20.2%	<0.001
Presence of musculoskeletal disorders	8.9%	10.6%	13.8%	18.9%	<0.001
Body mass index: underweight (<18.5)	6.0%	5.2%	6.1%	7.9%	<0.001
Alcohol intake: daily drinkers	25.3%	24.7%	21.5%	16.7%	<0.001
Smoking history: current smokers	14.0%	11.8%	10.1%	7.1%	<0.001
Frequency of meals: once or twice a day	7.5%	5.8%	6.0%	5.8%	0.002
Subjects with depression	17.7%	20.2%	24.8%	34.7%	<0.001
Subjects with poor cognitive function	9.7%	11.3%	16.1%	23.0%	<0.001
Subjects with poor self-rated health	13.8%	15.4%	17.9%	20.8%	<0.001
Subjects with no sense of purpose in life	10.7%	11.0%	11.2%	13.8%	<0.001
Subjects with poor IADL	7.7%	8.6%	10.6%	14.2%	<0.001
Engagement in volunteer groups	16.0%	20.1%	20.3%	16.9%	0.210
Engagement in sports groups	28.8%	29.7%	29.1%	21.7%	<0.001
Engagement in hobby groups	40.3%	45.6%	45.3%	38.7%	0.143
Engagement in cultural groups	15.4%	17.6%	18.0%	16.9%	0.035
Engagement in senior citizens’ clubs	5.9%	12.2%	18.6%	29.9%	<0.001
Engagement in neighborhood community associations	41.0%	42.8%	43.2%	36.4%	<0.001
Engagement in paid work	36.7%	25.6%	19.2%	9.4%	<0.001

^a^Cochran-Armitage test

Table 3 shows the gender-stratified characteristics of study participants. Females were significantly higher than males in the categories of “not being married,” “receiving national pension,” “presence of ophthalmologic disease,” “presence of musculoskeletal disorders,” “being underweight,” “participating in hobby groups,” “participating in cultural groups,” and “participating in senior citizens’ clubs” (Fisher’s exact test, *p* < 0.001). On the other hand, males were significantly higher than females in the categories of “daily drinkers,” “current smokers,” “eating less than 3 meals a day,” “having poor cognitive function,” “participating in volunteer groups,” “participating in sports groups,” “participating in neighborhood community associations,” and “participating in paid work” (Fisher’s exact test, *p* < 0.001). Regarding age, subjective economic situations, hypertension, and sensing a purpose in life, there was no difference between genders.

**Table 3:** Characteristics by gender among study participants (*n* = 17,680)

	Males (*n* = 8251)	Females (*n* = 9429)	*P* value^a^
Age: 75 years or older	35.2%	35.1%	0.925
Residential area: A City	49.5%	51.4%	0.011
Marital status: not married	13.1%	38.5%	<0.001
Subjective economic situations: poor	17.2%	17.2%	0.984
Pensions: National pension	15.3%	47.2%	<0.001
Presence of hypertension	40.1%	38.8%	0.076
Presence of ophthalmologic disease	13.6%	17.4%	<0.001
Presence of musculoskeletal disorders	7.2%	18.5%	<0.001
Body mass index: underweight (<18.5)	4.5%	8.0%	<0.001
Alcohol intake: daily drinkers	40.3%	5.8%	<0.001
Smoking history: current smokers	18.5%	3.8%	<0.001
Frequency of meals: once or twice a day	7.2%	5.6%	<0.001
Subjects with depression	23.7%	25.3%	0.013
Subjects with poor cognitive function	17.9%	12.9%	<0.001
Subjects with poor self-rated health	18.0%	16.2%	0.001
Subjects with no sense of purpose in life	12.1%	11.4%	0.181
Engagement in volunteer groups	19.9%	16.7%	<0.001
Engagement in sports groups	29.2%	25.4%	<0.001
Engagement in hobby groups	40.7%	43.7%	<0.001
Engagement in cultural groups	13.0%	20.4%	<0.001
Engagement in senior citizens’ clubs	15.5%	18.1%	<0.001
Engagement in neighborhood community associations	44.2%	37.7%	<0.001
Engagement in paid work	30.7%	15.5%	<0.001

^a^Fisher’s exact test

Table 4 shows the PR for poor IADL, stratified by age and gender group. Significant inverse association between volunteer groups and poor IADL was shown only in males and the impact was stronger on the old-old group than young-old group: among young-old males, the PRs of poor IADL were 0.74 (95% confidence interval [CI]:0.60–0.91) for infrequent participation and 0.95 (95% CI: 0.72–1.25) for frequent participation, and old-old males, scored 0.65 (0.49–0.85) and 0.63 (0.43–0.93), respectively, compared to those with non-participation. By contrast, significant inverse association between paid work and poor IADL was shown only in females, and infrequent participation was the only category that showed a significant relationship in both young-old (PR: 0.22, 95% CI: 0.05–0.90) and old-old (0.14, 0.02–0.96). Hobby groups were inversely associated with poor IADL, regardless of age, gender, and frequency. Sports groups showed a greater favorable effect on old-old group than the young-old group for both males and females. For senior citizens’ clubs, only the old-old who participated frequently had a significant inverse association with poor IADL. The matters involved in both males and females but showed greater positive impacts on old-old female group were cultural groups and neighborhood community associations. Overall, frequent participation had a stronger effect on old-old than young-old and in females than males.

**Table 4:** Adjusted prevalence ratios for poor IADL associated with the type and frequency of social participation, stratified by age and gender (reference: non-participation of each activity)

	Male young-old	Male old-old	Female young-old	Female old-old
	(*n* = 5348)	(*n* = 2903)	(*n* = 6119)	(*n* = 3310)
	PR^a^ (95% CI)	PR^a^ (95% CI)	PR^a^ (95% CI)	PR^a^ (95% CI)
Volunteer groups				
Infrequent^b^	**0.76 (0.62–0.94)** ^*^	**0.65 (0.50–0.86)** ^**^	0.53 (0.23–1.20)	0.81 (0.51–1.31)
Frequent^c^	0.96 (0.73–1.27)	**0.63 (0.43–0.94)** ^*^	0.40 (0.09–1.70)	0.71 (0.32–1.56)
Sports groups				
Infrequent^b^	**0.76 (0.62–0.93)** ^**^	**0.62 (0.44–0.86)** ^**^	0.14 (0.02–1.03)	**0.14 (0.02–0.95)** ^*^
Frequent^c^	0.85 (0.70–1.02)	**0.71 (0.55–0.93)** ^*^	**0.24 (0.10–0.59)** ^**^	**0.32 (0.18–0.55)** ^***^
Hobby groups				
Infrequent^b^	**0.75 (0.64–0.87)** ^***^	**0.64 (0.51–0.80)** ^***^	**0.18 (0.08–0.41)** ^***^	**0.59 (0.41–0.84)** ^**^
Frequent^c^	**0.81 (0.67–0.98)** ^*^	**0.70 (0.55–0.90)** ^**^	**0.18 (0.08–0.42)** ^***^	**0.27 (0.16–0.47)** ^***^
Cultural groups				
Infrequent^b^	**0.76 (0.59–0.99)** ^*^	**0.65 (0.48–0.89)** ^**^	**0.07 (0.01–0.52)** ^**^	**0.30 (0.17–0.55)** ^***^
Frequent^c^	0.83 (0.54–1.30)	0.85 (0.53–1.35)	0.36 (0.09–1.47)	**0.20 (0.07–0.60)** ^**^
Senior citizens’ clubs				
Infrequent^d^	1.16 (0.91–1.47)	0.89 (0.69–1.14)	0.35 (0.09–1.41)	0.83 (0.59–1.18)
Frequent^e^	0.91 (0.67–1.25)	**0.65 (0.49–0.86)** ^**^	0.56 (0.23–1.37)	**0.52 (0.36–0.76)** ^**^
Neighborhood community associations				
Infrequent^d^	0.90 (0.78–1.02)	**0.74 (0.62–0.88)** ^**^	**0.40 (0.24–0.67)** ^***^	**0.62 (0.44–0.88)** ^**^
Frequent^e^	**0.71 (0.56–0.90)** ^**^	0.76 (0.56–1.02)	0.46 (0.18–1.19)	**0.47 (0.27–0.84)** ^*^
Paid work				
Infrequent^f^	1.01 (0.86–1.20)	0.96 (0.73–1.26)	**0.21 (0.05–0.87)** ^*^	0.14 (0.02–0.99)*
Frequent^g^	1.01 (0.86–1.18)	1.00 (0.74–1.35)	0.56 (0.23–1.36)	1.06 (0.46–2.46)

*CI* confidence interval, *IADL* instrumental activities of daily living, *PR* prevalence ratio

Young-old means aged 65–74, and old-old means aged 75 or over

Bold values highlight statistical significance

^*^ *p* < 0.05, ^**^ *p* < 0.01, ^***^ *p* < 0.001

^a^Adjusted for age, residential area, marital status, subjective economic situations, pensions, chronic diseases, body mass index, alcohol, smoking, dietary habit, depression, cognitive function, self-rated health, and sensing a purpose in life. ^b^Weekly or more. ^c^Monthly or less. ^d^Monthly or more. ^e^Yearly. ^f^Four times or more per week. ^g^Three times or less per week

## Discussion

From the overall tendency, preferable impact given to elderly people’s IADL from SP is stronger on females than males and the same can be said to those who are in the old-old category than the young-old category. Regarding the mechanism of this study’s results, please refer to previous studies regarding SP provides good impact on IADL, and females have stronger relationships with IADL than males [14, 15]. In this study, we solely report the mechanism based on the newly obtained results.

First, among only females, low frequency paid work showed a significant relationship with independent IADL. Some prior studies demonstrated that paid work in the elderly produced a good effect on physical functioning [19, 29]. Minami et al. reported that there is a relationship between continuation of paid work and maintenance of higher-level functional capacity [29]. However, the interpretation of the results in the Minami study requires careful attention because higher-level functional capacity includes social role, which is a similar concept to SP, and no analyses were conducted on each gender. In another study [19], working local dwelling elderly people showed a significant decrease to the risk of deteriorating BADL among males during their 8 years of follow-up period, though females had no association between paid work and BADL. The results of the Fujiwara study [19] are inconsistent with those of the present study. One reason is the difference of outcome indexes and in addition, because employment status in the Fujiwara study was evaluated 25 years ago, in 1991. Since then, systems of elderly employment and pension have drastically changed, and the public nursing-care insurance system has been introduced, so elderly people’s work environment and self-care may differ greatly between the time the former study was conducted in 1991 and our study was conducted in 2014. In a recent study [30], the researchers examined elderly people who visited local employment support center to find out the reasons each gender were looking for jobs. A significant number of females answered that they were looking for work for their well-being or SP. On the other hand, more males replied that they wanted to pay debts or were acting on the recommendations by family members. That means males participate in paid work with a more passive attitude than females and that may be why it is difficult for males to receive positive influences from paid work. Additionally, a prospective study reported that compared to voluntary retirees, older workers with good quality jobs had better health effects, but those with poor quality jobs had adverse health effects [20]. Our results regarding the relationship between paid work and IADL among females showed a significant positive association only for infrequent participation but not for frequent participation. These findings suggest that more participation in paid work by the elderly does not mean better impact on their health, and job quality may play an important role in the health of older workers.

Second, we examined the results of volunteer groups. Morrow-Howell has argued that volunteering can produce social integration, altruism, a purpose in life, and a positive emotional state, and these aspects are considered as a mechanism responsible for the beneficial effect of volunteering on health [31]. In particular, an altruistic nature is present only in volunteering and not in other social activities [32]. Additionally, he has pointed out that “volunteering is socially valued, publicly recognized, and more discretionary than working” [31]. In Japan, more adult males are assuming management and specialist positions as full-time workers than adult females [33]. It is considered that adult males gain greater societal roles and motivations in life from their work than adult females [34]. Elderly people tend to retire or even if they continue working, they work irregularly [29, 35] meaning males can no longer earn societal roles and motivation. Therefore, we consider they feel they make a valuable contribution to society through their volunteer activities [31] and that is why favorable impact on IADL from volunteer activities is shown only in male subjects.

Finally, we would like to discuss why the old-old group receives stronger favorable impact on their IADL from SPs than the young-old. Because IADL functioning involves more-multifaceted and cognitively challenging behaviors compared to BADL performance, the procedure for IADL makes not only functional mobility but also cognitive ability necessary [36]. Physical and cognitive functions diminish along with aging and it should be noted, the speed of deterioration accelerates in the old-old group [16, 37]. Elderly people’s physical activities can prevent diminishment of physical function [38] and cognitive function [39], while their mental and social activities can maintain physical function [2, 4] and cognitive function [5, 6]. Furthermore, the longer people live the higher chance they have to experience the deaths of people close and beloved to them, which may trigger deterioration of elderly people’s physical function [40]. However, it is known that social support can protect against functional deterioration caused by spousal loss [41]. SPs can increase the amount of physical activity, and give cognitive stimulation and social support that prevents functional decline during the elderly period, and we consider such impact to be greater on the old-old group than the young-old group. This study also supports the above tentative theory as our survey noted sports groups for practicing physical exercises, cultural groups for intellectually stimulating activities, and senior citizen’s clubs for exercises and brain function trainings, all of which showed a greater relationship with independent IADL for the old-old group than the young-old group.

We would like to discuss about the generalization of this study. Among this study population, 30.7% of males and 15.5% of females were engaged in paid work (see Table 3). It is reported that the year our survey was taken, 2014, the employment rate for people 65 years or older in Japan was 29.3% for males and 14.3% for females [33]. Because this study limited its subjects to those with autonomous BADL, the employment rate is pushed higher compared to all senior citizens, but we consider these numbers to somewhat show the average condition of elderly people’s employment rate. Not only for SPs including paid work, but it is important for IADL to consider neighborhood environment, for example, whether accessibilities to public transportation is good or whether SPs are held near participants’ homes [13, 23]. Therefore, it requires caution when the researchers generalize the results of this study in rural areas and metropolitan cities.

There are limitations in this study. First, because it is a cross-sectional study, we cannot identify causal correlations. It is necessary to conduct follow-up studies to confirm whether frequent SP at baseline is related to maintaining IADL. Second, the collection rate of the questionnaire is not high. In this study, we examined the differences of basic attributes between those who submitted questionnaires and those who didn’t (see Additional file 6). More people in the higher age group and those with lower physical function didn’t submit the questionnaire, which means those who are not conducting SP and those with diminished IADL can be selectively eliminated. This selection bias is considered to lead the results of the study to underestimation. Third, because both independent and dependent variables are based on self-rating, it is possible that the results are overestimated by a common method bias [42]. On the other hand, it is considered that misclassification brought by self-rating may bias the degree of influence from SP to IADL toward null value [43].

## Conclusions

Although there are the limitations stated above, this study is based on complete census data from 2 local governments, and we could earn useful knowledge for the realization of a healthier aging society. The results of this study reconfirmed SP is significantly related to independent IADL among community dwelling elderly people. Additionally, it was revealed this significant relationship differs depending on the type and frequency of participation as well as gender and age group. When one recommends SP to community dwelling elderly people for the purpose of maintaining their IADL, we should suggest proper type and frequency of SP activities, taking into consideration their gender and age.

## Additional files

Additional file 1: Table S1. The Tokyo Metropolitan Institute of Gerontology Index of Competence. (PDF 64 kb)

Additional file 2: Table S2. The distribution of type and frequency of social participation. (PDF 68 kb)

Additional file 3: Table S3. The distribution of social participation of subjects with and without poor instrumental activities of daily living (IADL) by age and gender. (PDF 88 kb)

Additional file 4: Table S4. Characteristics of analyzed subjects’ responses to the questionnaire. (PDF 68 kb)

Additional file 5: Sample size approximation. (PDF 88 kb)

Additional file 6: Table S6. Basic attributes of subjects who submitted the questionnaire. (PDF 68 kb)

## Abbreviations

BADL: Basic activities of daily living
IADL: Instrumental activities of daily living
SP: Social participation
