# Supplementary material for: Age and gender differences in the association between social participation and instrumental activities of daily living among community-dwelling elderly
Source: BMC Geriatr. 2017 Apr 28;17:99. doi: 10.1186/s12877-017-0491-7 (PMC5410028; doi:10.1186/s12877-017-0491-7)
Supplement: Supplementary file 1 — The Tokyo Metropolitan Institute of Gerontology Index of Competence. (PDF 64 kb) [file 12877_2017_491_MOESM1_ESM.pdf]

Additional file 1: Table S1. The Tokyo Metropolitan Institute of Gerontology Index of Competence

Subscales	Answers	
Instrumental Activities of Daily Living (IADL)		
1 Can you use public transportation (bus or train) by yourself ?	1. Yes	0. No
2 Are you able to shop for daily necessities ?	1. Yes	0. No
3 Are you able to prepare meals by yourself ?	1. Yes	0. No
4 Are you able to pay bills ?	1. Yes	0. No
5 Can you handle your own banking ?	1. Yes	0. No
Intellectual Activity		
6 Are you able to fill out forms for your pension ?	1. Yes	0. No
7 Do you read newspapers ?	1. Yes	0. No
8 Do you read books or magazines ?	1. Yes	0. No
9 Are you interested in news stories or programs dealing with health ?	1. Yes	0. No
Social Role		
10 Do you visit the homes of friends ?	1. Yes	0. No
11 Are you sometimes called on for advice ?	1. Yes	0. No
12 Are you able to visit sick friends ?	1. Yes	0. No
13 Do you sometimes initiate conversations with young people ?	1. Yes	0. No
