# Supplementary material for: Age and gender differences in the association between social participation and instrumental activities of daily living among community-dwelling elderly
Source: BMC Geriatr. 2017 Apr 28;17:99. doi: 10.1186/s12877-017-0491-7 (PMC5410028; doi:10.1186/s12877-017-0491-7)
Supplement: Supplementary file 2 — The distribution of type and frequency of social participation. (PDF 68 kb) [file 12877_2017_491_MOESM2_ESM.pdf]

Additional file 2: Table S2. The distribution of type and frequency of social participation

		n	(%)	Category of frequency
Volunteer groups	Non-participation	14,464	(81.8)	Non-participation
	Several times a year	1102	(6.2)	Infrequent
	Several times a month	1085	(6.1)	
	Once a week	407	(2.3)	Frequent
	Several times a week	406	(2.3)	
	Four or more times a week	216	(1.2)	
Sports groups	Non-participation	12,874	(72.8)	Non-participation
	Several times a year	679	(3.8)	Infrequent
	Several times a month	953	(5.4)	
	Once a week	1021	(5.8)	Frequent
	Several times a week	1410	(8.0)	
	Four or more times a week	743	(4.2)	
Hobby groups	Non-participation	10,204	(57.7)	Non-participation
	Several times a year	1388	(7.9)	Infrequent
	Several times a month	2781	(15.7)	
	Once a week	1427	(8.1)	Frequent
	Several times a week	1336	(7.6)	
	Four or more times a week	544	(3.1)	
Cultural groups	Non-participation	14,689	(83.1)	Non-participation
	Several times a year	1022	(5.8)	Infrequent
	Several times a month	1158	(6.5)	
	Once a week	448	(2.5)	Frequent
	Several times a week	256	(1.4)	
	Four or more times a week	107	(0.6)	
Senior citizens' clubs	Non-participation	14,697	(83.1)	Non-participation
	Several times a year	1247	(7.1)	Infrequent
	Several times a month	1181	(6.7)	Frequent
	Once a week	191	(1.1)	
	Several times a week	280	(1.6)	
	Four or more times a week	84	(0.5)	
Neighborhood community associations	Non-participation	10,480	(59.3)	Non-participation
	Several times a year	5576	(31.5)	Infrequent
	Several times a month	1197	(6.8)	Frequent
	Once a week	187	(1.1)	
	Several times a week	160	(0.9)	
	Four or more times a week	80	(0.5)	
Paid work	Non-participation	13,679	(77.4)	Non-participation
	Several times a year	278	(1.6)	Infrequent
	Several times a month	272	(1.5)	
	Once a week	280	(1.6)	
	Several times a week	973	(5.5)	
	Four or more times a week	2198	(12.4)	Frequent
