# Supplementary material for: Age and gender differences in the association between social participation and instrumental activities of daily living among community-dwelling elderly
Source: BMC Geriatr. 2017 Apr 28;17:99. doi: 10.1186/s12877-017-0491-7 (PMC5410028; doi:10.1186/s12877-017-0491-7)
Supplement: Supplementary file 3 — The distribution of social participation of subjects with and without poor instrumental activities of daily living (IADL) by age and gender. (PDF 88 kb) [file 12877_2017_491_MOESM3_ESM.pdf]

Additional file 3: Table S3. The distribution of social participation of subjects with and without poor instrumental activities of daily living (IADL) by age and gender

	Male young-old		Male old-old		Female young-old		Female old-old	
	Independent IADL, *n*	Poor IADL, *n*	Independent IADL, *n*	Poor IADL, *n*	Independent IADL, *n*	Poor IADL, *n*	Independent IADL, *n*	Poor IADL, *n*
Volunteer groups								
Non-participation	3572	740	1825	476	4898	118	2564	271
Yearly	348	54	201	25	326	4	136	8
Monthly	287	31	152	21	413	2	170	9
Weekly	109	22	64	9	148	1	51	3
2-3 times a week	100	10	67	5	152	1	69	2
≥4 times a week	61	14	51	7	56	0	26	1
Sports groups								
Non-participation	3019	665	1696	458	4265	120	2369	282
Yearly	337	43	130	15	113	0	41	0
Monthly	396	52	146	17	251	1	89	1
Weekly	203	33	96	14	490	2	179	4
2-3 times a week	307	49	196	25	589	1	237	6
≥4 times a week	215	29	96	14	285	2	101	1
Hobby groups								
Non-participation	2559	594	1336	402	3198	115	1749	251
Yearly	532	86	240	40	335	2	143	10
Monthly	699	85	335	38	1134	4	466	20
Weekly	272	46	165	31	610	3	292	8
2-3 times a week	269	45	186	19	536	0	277	4
≥4 times a week	146	15	98	13	180	2	89	1
Cultural groups								
Non-participation	3912	801	1974	493	4723	123	2382	281
Yearly	269	33	172	16	394	0	135	3
Monthly	170	20	133	20	514	1	293	7
Weekly	74	12	43	6	192	1	117	3
2-3 times a week	32	3	27	4	128	0	62	0
≥4 times a week	20	2	11	4	42	1	27	0
Senior citizens’ clubs								
Non-participation	4033	779	1723	441	5281	119	2084	237
Yearly	232	56	259	54	290	2	325	29
Monthly	115	24	249	33	292	5	438	25
Weekly	34	1	39	7	48	0	60	2
2-3 times a week	44	7	70	8	66	0	84	1
≥4 times a week	19	4	20	0	16	0	25	0
Neighborhood community associations								
Non-participation	2439	535	1263	365	3536	105	1988	249
Yearly	1529	269	817	136	2034	17	740	34
Monthly	376	47	189	25	337	4	208	11
Weekly	63	7	31	8	45	0	33	0
2-3 times a week	37	8	37	7	33	0	38	0
≥4 times a week	33	5	23	2	8	0	9	0
Paid work								
Non-participation	2737	547	1969	463	4764	119	2792	288
Yearly	102	19	40	12	75	0	29	1
Monthly	88	15	40	7	93	0	29	0
Weekly	98	14	42	8	91	1	26	0
2-3 times a week	451	90	83	15	290	1	43	0
≥4 times a week	1001	186	186	38	680	5	97	5
