# Supplementary material for: Age and gender differences in the association between social participation and instrumental activities of daily living among community-dwelling elderly
Source: BMC Geriatr. 2017 Apr 28;17:99. doi: 10.1186/s12877-017-0491-7 (PMC5410028; doi:10.1186/s12877-017-0491-7)
Supplement: Supplementary file 4 — Characteristics of analyzed subjects’ responses to the questionnaire. (PDF 68 kb) [file 12877_2017_491_MOESM4_ESM.pdf]

Additional file 4: Table S4. Characteristics of analyzed subjects' responses to the questionnaire

Item	Category	n	(%)
Residential area	A City	8937	(50.5)
	B City	8743	(49.5)
Marital status	Currently married	12,792	(72.4)
	Not married	4715	(26.7)
	Missing	173	(1.0)
Subjective economic situations	Very well set	912	(5.2)
	Somewhat well set	5626	(31.8)
	Somewhat poor	6982	(39.5)
	Poor	3045	(17.2)
	Missing	1115	(6.3)
Pensions	National pension	5706	(32.3)
	Employees' pension	8892	(50.3)
	Mutual aid association pension	1581	(8.9)
	Other	762	(4.3)
	Missing	739	(4.2)
Chronic diseases under medical treatment	Hypertension	6969	(39.4)
	Ophthalmologic disease	2762	(15.6)
	Musculoskeletal disorders	2332	(13.2)
	Diabetes mellitus	2214	(12.5)
	Heart disease	1787	(10.1)
	Digestive system disease	1721	(9.7)
	Otological disease	1383	(7.8)
	Urogenital disease	1370	(7.7)
	Chronic respiratory disease	926	(5.2)
	Cancer	628	(3.6)
	Cerebrovascular disease	544	(3.1)
Body mass index	Normal (18.5-<25.0)	12,165	(68.8)
	Underweight (<18.5)	1128	(6.4)
	Overweight (≥25.0)	3619	(20.5)
	Missing	768	(4.3)
Alcohol intake	Nondrinkers	6439	(36.4)
	Social drinkers	4127	(23.3)
	Occasional drinkers	2577	(14.6)
	Daily drinkers	3870	(21.9)
	Missing	667	(3.8)
Smoking history	Never-smokers	9968	(56.4)
	Ex-smokers	5094	(28.8)
	Current smokers	1892	(10.7)
	Missing	726	(4.1)

Additional file 4: Table S4. Continued.

Item	Category	n	(%)
Dietary habit (frequency of meals)	Three or more times a day	16,213	(91.7)
	Once or twice a day	1119	(6.3)
	Missing	348	(2.0)
Depression	Absent	12,761	(72.2)
	Present	4342	(24.6)
	Missing	577	(3.3)
Cognitive function	Intact	14,729	(83.3)
	Poor	2695	(15.2)
	Missing	256	(1.4)
Self-rated health	Very good	1918	(10.8)
	Good	11,921	(67.4)
	Poor	2334	(13.2)
	Very poor	679	(3.8)
	Missing	828	(4.7)
Having a purpose in life	Yes (having)	15,132	(85.6)
	No (not having)	2070	(11.7)
	Missing	478	(2.7)
