# Supplementary material for: Age and gender differences in the association between social participation and instrumental activities of daily living among community-dwelling elderly
Source: BMC Geriatr. 2017 Apr 28;17:99. doi: 10.1186/s12877-017-0491-7 (PMC5410028; doi:10.1186/s12877-017-0491-7)
Supplement: Supplementary file 5 — Sample size approximation. (PDF 88 kb) [file 12877_2017_491_MOESM5_ESM.pdf]

Additional file 5. Sample size approximation

Based on prior studies, among male elderly, the proportion of poor IADL is assumed to be 0.15 in the working/participation group and 0.20 in non-working/non-participation group. Among female elderly, the proportion of poor IADL is assumed to be 0.05 in the working/participation group and 0.10 in non-working/non-participation group.

Regarding paid work, among males, the ratio of the non-working group to the working group is assumed to be 2.3, based on the Annual Report on the Labor Force Survey 2015 that the rate of employment in the elderly aged 65 over was 30.3%. Group sample sizes of 689 in the working group and 1585 in the non-working group achieved 80% power to detect a difference between the group proportions of 5%. Among females, the ratio of the non-working group to the working group is assumed to be 5.7, based on the Annual Report on the Labor Force Survey 2015 that the rate of the elderly's employment was 15.0%. Group sample sizes of 297 in the working group and 1693 in the non-working group achieved 80% power to detect a difference between the group proportions of 5%.

Regarding social group activities, according to the Annual Report on the Aging Society 2016, 61.0% of older people aged 60 and older participated in some types of social group activity. Our previous study also reported that the percentage of

community-dwelling elderly participating in any type of social group was 67.6%, and there was no gender difference in participation level. Therefore, we assumed the ratio of subjects without group participation to those with group participation was 0.5 for both genders. Among males, group sample sizes of 699 in the non-participation group and 1397 in the participation group achieved 80% power to detect a difference between the group proportions of 5%. Among females, group sample sizes of 341 in the non-participation group and 682 in the participation group achieved 80% power to detect a difference between the group proportions of 5%. Among both genders, the test statistic used was the two-sided Chi square Test. The significance level of the test is 0.05.

As explained above, we believe our study had an adequate design of needed sample size.
