# Supplementary material for: Age and gender differences in the association between social participation and instrumental activities of daily living among community-dwelling elderly
Source: BMC Geriatr. 2017 Apr 28;17:99. doi: 10.1186/s12877-017-0491-7 (PMC5410028; doi:10.1186/s12877-017-0491-7)
Supplement: Supplementary file 6 — Basic attributes of subjects who submitted the questionnaire. (PDF 68 kb) [file 12877_2017_491_MOESM6_ESM.pdf]

Additional file 6: Table S6. Basic attributes of subjects who submitted the questionnaire

	No. distributed	No. of submission	Response rate	*P* value
Age				
Aged 65-69	9869	6570	66.6%	0.002[a]
Aged 70-74	8902	6398	71.9%	
Aged 75-79	6296	4550	72.3%	
Aged 80-84	4148	2984	71.9%	
Aged 85-89	2371	1589	67.0%	
Aged 90+	1238	754	60.9%	
Level of care needed				
None (independent)	27,988	19,968	71.3%	<0.001[a]
Requiring help 1	790	599	75.8%	
Requiring help 2	703	509	72.4%	
Long-term care level 1	890	534	60.0%	
Long-term care level 2	861	492	57.1%	
Long-term care level 3	612	326	53.3%	
Long-term care level 4	557	248	44.5%	
Long-term care level 5 (totally dependent)	423	169	40.0%	
Gender				
Males	14,392	10,060	69.9%	0.150[b]
Females	18,432	12,785	69.4%	

[a]Cochran-Armitage test, [b]Fisher's exact test.
